# Blockage of Kv1.3 regulates macrophage migration in acute liver injury by targeting δ-catenin through RhoA signaling

**DOI:** 10.7150/ijbs.38950

**Published:** 2020-01-14

**Authors:** Baoming Wu, Jun-da Liu, Erbao Bian, Wei Hu, Cheng Huang, Xiaoming Meng, Lei Zhang, Xiongwen Lv, Jun Li

**Affiliations:** 1 School of Pharmacy, Anhui Key Laboratory of Bioactivity of Natural Products, Anhui Medical University, Hefei 230032, China.; 2 The Key Laboratory of Anti-inflammatory and Immune Medicine, Anhui Medical University, Ministry of Education, Hefei 230032, China.; 3 Institute for Liver Diseases of Anhui Medical University, ILD-AMU, Anhui Medical University, Hefei 230032, China.; 4 Anhui Institute of Innovative Drugs, Anhui Medical University, Hefei 230032, China; 5 The first affiliated hospital of Anhui medical university, Hefei 230032, China; 6 The second affiliated hospital of Anhui medical university, Hefei 230032, China

**Keywords:** Kv1.3, Margatoxin, Acute liver injury, Migration, δ-catenin, RhoA

## Abstract

**Background**: Activation of macrophages and infiltration are key events in acute liver injury (ALI). Kv1.3 plays an important role in regulating immunologic functions of macrophages and is extensively recognized as a potential ion channel for immunological diseases.

**Objective**: We hypothesized that blockage of Kv1.3 may influence ALI by inhibiting macrophages infiltration in damaged liver tissues.

**Methods**: Margatoxin was administered into the peritoneal cavity of ALI mice. The impact of this treatment on ALI and macrophage migration *in vivo and in vitro* was determined using immunohistochemistry, transwell migration, and wound healing assays.

**Results**: MgTX treatment alleviated ALI in mice, as evidenced by reduced macrophage infiltration in liver tissues and lower serum levels of liver ALT and AST. RNA-seq profiling analysis showed that the most obvious change by MgTX treatment was downregulation of δ-catenin, a protein known to be associated with macrophage migration. The effect of MgTX on macrophage migration and involvement of δ-catenin was confirmed by transwell and wound healing assays. Overexpression of δ-catenin in RAW264.7 cells promoted migration, an event that was suppressed upon silencing of δ-catenin. Mechanistically, the expression of RhoA was regulated by the overexpression or knockdown of δ-catenin.

**Conclusion**: These findings suggest a role for blockage of Kv1.3 channel in macrophage migration and reveal a new target in the treatment of ALI.

## Introduction

Acute liver failure (ALF) is caused by serious hepatic injury with abnormal function of hepatocyte and leading to different clinical syndrome, such as coagulopathy, encephalopathy, and circulatory dysfunction. ALF is related with high mortality in liver diseases, ranging from 30 to 80%^1^. As crucial immunocyte of the hepatic innate immune system, Kupffer cells (KCs) are recognized to play a key role in response to lipopolysaccharide (LPS) exposure. under treatment with LPS, KCs secrete different inflammatory cytokines, including interleukin-1 (IL-1), interleukin-6 (IL-6), monocyte chemoattractant protein-1 (MCP-1), and tumor necrosis factor- α (TNF-α). these proinflammatory cytokines can induce hepatocyte death and ultimately resulting in ALF 2. Two types of macrophages exert their immunological functions in liver diseases, the resident KCs and the circulating macrophages that differentiate from monocytes. Circulating monocytes are important in complementing hepatic macrophage pool for homeostasis, and hepatic metabolism or toxic impairment leads to numerous infiltration of monocyte-derived macrophages into the liver^3^**.** Infiltration of monocytes plays a crucial role in the progression of neuroinflammatory diseases, inhibit monocytes infiltrate into injury CNS recognized as a potential therapeutic strategy 4. Similarly, infiltration of monocytes may be a key event in the development of liver fibrosis, thus, targeting monocyte migration during liver injury may be an important strategy.

Kv1.3 belongs to a subtype of voltage-gated potassium channel, which has six transmembrane domains (S1-S6), including a voltage sensor (S4) and a pore-forming region^5^. Margatoxin (MgTX) contains 39 amino-acid-long peptides isolated from the venom of the scorpion *Centruroidesn margaritatus*. It is stabilized by three disulfide bridges with a molecular weight of 4185. Margatoxin has a special affinity for Kv1.3 and is a high selective inhibitor for Kv1.3 channel^6^-^7^. Previous reports have shown that Kv1.3 can enhance migration on vascular smooth muscle cell 8. Nonetheless, most studies on the Kv1.3 channel have focused on its role in T cells, however, the role of Kv1.3 channel in targeting macrophage function has drawn little attention. In the present study, we aimed to study the effects and underlying mechanism of Kv1.3 in modulating macrophage migration in acute liver injury.

We established an ALI mouse model using LPS+D-GAIN (D-galactosamine) and treated these mice with MgTX injection in the peritoneal cavity. The effect of MgTX on macrophage migration into liver tissues and alleviation of liver injury was investigated. RNA-seq analysis showed that MgTX inhibited macrophage migration through a decrease in δ-catenin RNA expression. Furthermore, we found that δ-catenin could modulate the expression of its downstream protein RhoA to inhibit macrophage migration and, ultimately, alleviate ALI. Our proof-of-concept study suggests that inhibition of Kv1.3 is a potential therapeutic strategy for acute liver diseases.

## Materials and Methods

### LPS+D-GAIN acute liver injury model

Animal protocols were approved by the Animal Care and Use Committee of Anhui Medical University, Hefei, China. C57BL/6 (20 ± 2 g) male mice were purchased from the Experimental Animal Center of Anhui Medical University for the LPS+D-GAIN model 2. ALI was established by intraperitoneal injection of LPS (3 μg/kg) + D-GAIN (200 mg/kg, Sigma Aldrich, St Louis, MO, USA). Animals in the experimental group were injected intraperitoneally with 100 nM Margatoxin, while control mice were treated with saline.

### Serum liver enzyme activity assay

The levels of alanine aminotransferease (ALT) and aspartate aminotransferase (AST) in serum were measured using ALT and AST activity assay kits (C009-2 and A110-1, Jiancheng, Nanjing, China). The absorbance was measured at 510 nm with a Multiskan MK3 microplate reader (Thermo Fisher, USA).

### ELISA

Serum was extracted from blood using centrifugation (3,000 rpm; 10 min) and stored at -80˚C for subsequent use. The levels of TNF-α was determined in mouse serum using ELISA kits according to the manufacturer's protocol. mouse TNF-α ELISA kit (cat. no. JYM028Mo) was used and all purchased from Jiyinmei biotechnology co., Wuhan.

### Histopathology

The middle portion of the left hepatic lobe was sectioned and fixed in 4% paraformaldehyde for 48 h, then the tissues were embedded in paraffin, and 5 μm thick slices were stained with hematoxylin - eosin (HE) for morphological analysis, Masson's trichrome for evaluation of collagen expression.

### Cell culture

The RAW264.7 cell lines was purchased from the Type Culture Collection of the Chinese Academy of Sciences (Shanghai, China). Cells were cultured in Dulbecco's-modified Eagle's medium (DMEM; Gibco, USA) supplemented with fetal bovine serum (10%, Gibco, USA), penicillin and streptomycin (1%) and cultured at 37°C in a 5% CO_2_ incubator.

### Flow cytometry

To identify effects of MgTX on monocytes migration in acute liver injury, flow cytometry analysis was performed. Fresh liver was dissociated into single-cell suspensions by combining mechanical dissociation according liver dissociation kit (miltenyi biotec, 130105807, USA). Cells were incubated away from light with fluorescent-labeled anti-mouse antibodies for 15 min at 4°C, then cells were washed in PBS (1ml) and centrifugated (300 g) for 5 min, 300 µl resuspended at ~5×106/ml after 1 h for flow cytometry (BD Accuri C6, USA). FlowJo version 10 software (BD Biosciences, CA, USA) was used to analyze the data. Antibodies used in this experiment were as follows: anti-CD45-perCP-Cy5.5 (cat. no. 561869, BD), anti-Ly6GLy6C-PE (cat. no. 561084, BD), anti-CD11b-FITC (cat. no. 561688, BD), and CD192-AF647 (cat. no. 150603, BioLegend).

### RNA-seq and data processing

RAW264.7 cells grown in DMEM medium and subjected to 10 nM MgTX for 12 h treatment were collected. Untreated cells served as control. The total RNA was extracted using standard protocols and high-throughput sequencing was detected on illumina HiSeq 2000 platform in GENE DENOVO (Guangzhou, China). Raw sequences containing adapters and low-quality bases were filtered and mapped to the *C. reinhardtii* reference genome, using the TopHat2 aligner, dates can be got from Phytozome 11 database (JGI). Bioconductor edger was used for differential expression analysis of RNA-seq expression profiles. Gene absolute values of p ≤0.05 and log2 (fold change) ≥1 were set as thresholds of DGEs (differentially expressed genes). After analysis, the DGEs were subjected to enrichment analysis of GO functions and KEGG pathways.

### Small interfering RNA and plasmid transfection

To overexpress and downregulate the expression of δ-catenin, RAW264.7 cells were transfected with plasmid or small interfering RNA (siRNA), respectively, using lipofectamine 2000 reagent (Invitrogen, USA) following to the manufacturer's instructions. SiRNA oligonucleotides against δ-catenin, overexpression plasmid was designed and constructed by Shanghai GenePharma Corporation. RAW264.7 cell lines were transfected with siRNA or plasmids in opti-MEM culture medium (Invitrogen, USA). After 6 h transfection, the opti-MEM culture medium was changed to DMEM, and cells were cultured at 37°C in a 5% CO_2_ incubator for 12 h.

### Transwell migration assay

Transwell chambers (Corning, Tewksbury, MA, USA, 8.0μm) were pre-wet with serum-free DMEM for 30 min prior to use. The number of cells is adjusted to a concentration of 1 × 10^5^ /well using serum-free medium, and then the drug (MgTX) or an equal amount of vehicle control (distilled water) was added to the cell suspension. Next, 200 μL cell suspension was placed with serum-free medium into the upper chamber, 600 μL same medium with 10% FBS was placed in the lower chamber. After culturing for 16-18 h at 37°C in 5% CO_2_ incubator, the upper chambers were removed from the Transwell system and fixed in methanol for 20 min. Cells on the upper side of the filters were removed with cotton-tipped swabs and the filters washed with 0.01M PBS. Cells on the lower side of the filters were stained with 0.5% crystal violet in PBS for 15 min. The image of the cells was counted under a microscope, and the number of migrating cells was recorded with ImageJ software. Each treatment was performed in triplicate and repeated for at least three times.

### Wound scratch assay

Wound healing assay was performed as previously described 9. Transfected and control cells were grown and placed in a 6-well plate. After 12 h, cells were scratched using sterile pipette tip. Each wounded area was photographed after scratching at 0 and 12 h. The wound healing capability was measured by counting the percentage of cell coverage to covering the scratch area after 12 h.

### Total RNA extraction and quantitative real-time PCR (RT-qPCR)

Total RNA was extracted from RAW264.7 cell lines using TRIzol reagent (Invitrogen, USA). Using ThermoScript RT-qPCR synthesis kit (Fermentas, USA) to synthesized cDNA following to the manufacturer's protocol. Real-time quantitative PCR analysis for mRNA of *δ-catenin* and *GAPDH* were performed with ThermoScript RT-qPCR kits (Fermentas, USA). GAPDH was used to normalize input RNA. Relative RNA expression level was calculated following the standard 2^-ΔΔCt^ method. each experiment was performed in triplicate and repeated for at least three times.

### Western Blotting

RAW264.7 cells were lysed with protein extraction solution (Beyotime, China). Protein concentration of each sample was measured by NanoDrop 2000 Spectrophotometer (Thermo scientific, USA). Samples were performed using electrophoresis on SDS-PAGE and transferred onto PVDF membrane (Millipore Corp, Billerica, MA, USA). After PVDF membrane was sealed with nonfat 5% milk for 2 h, then followed by incubation with primary antibodies at 4°C overnight. The concentration of primary antibodies against δ-catenin (Bioss, China) and β-actin were used at 1:500. Subsequently, the PVDF membranes were washed three times with TBST, followed by incubation with secondary antibodies (1:10,000) at room temperature for 1.5 h. After washing three times with TBST, protein blots were observed using ECL-chemiluminescent kit (ECL-plus, Thermo Scientific, USA).

### Statistical Analysis

Statistical analysis was performed with the Statistical Package for Social Sciences v.13.0 (SPSS Inc., Chicago, IL, USA,). Each experiment was performed at least three times and the date are presented as the mean ± SD. Statistical differences of the results were analyzed by one-way ANOVA. Dates were considered statistically significant when* P* < 0.05.

## Results

### 1. MgTX attenuates acute liver injury *in vivo*

**Figure 1:**
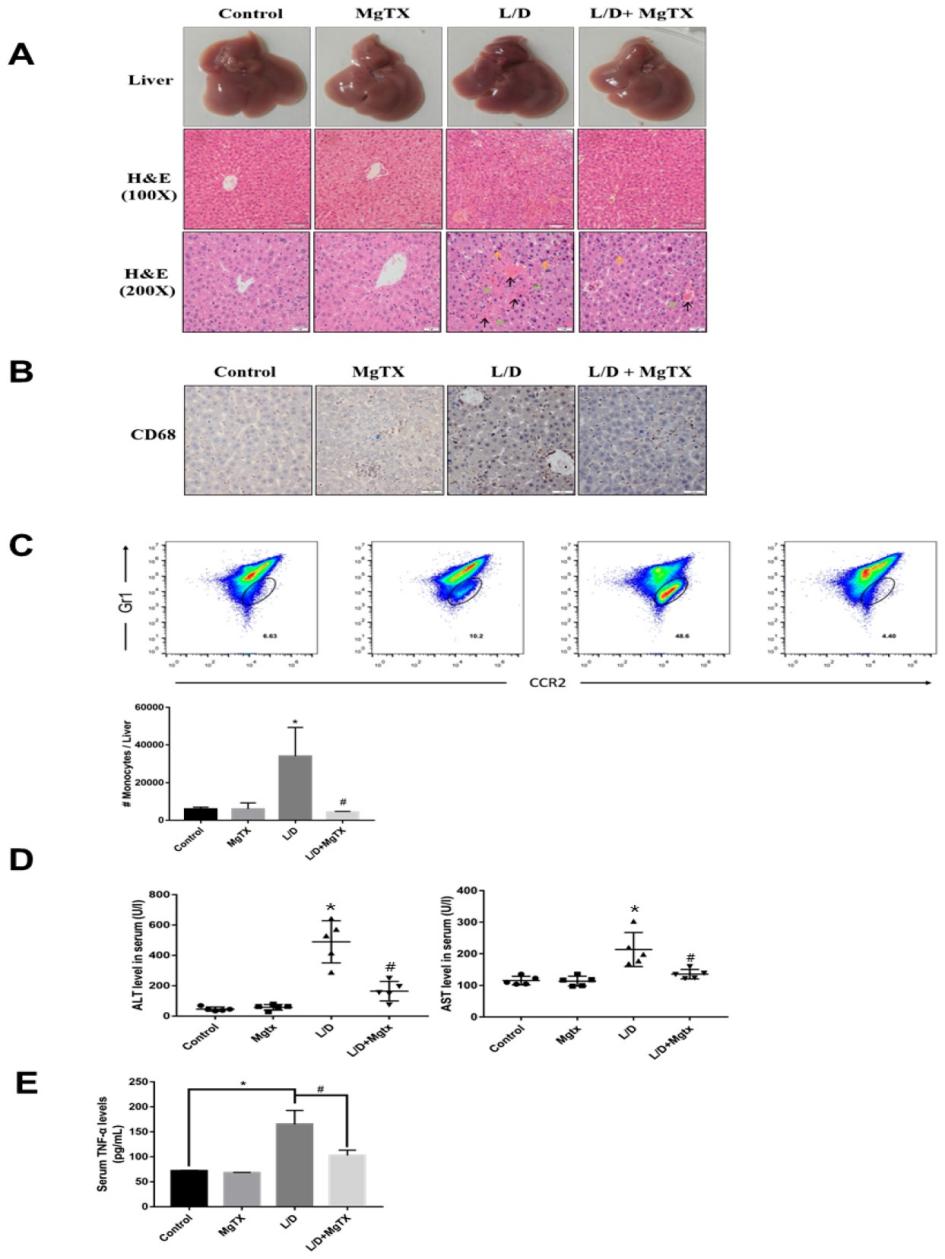
MgTX protects mice from LPS+D-GAIN (L/D)-induced liver injury. Mice were administered MgTX (100 nM) intraperitoneally in combination with LPS+D-GAlN or saline as the control. **(A)** MgTX attenuated ALI in LPS+D-GAIN model (n= 10 for each group). Macroscopic appearance of representative liver samples and H&E staining of the different groups (as indicated) at 12 h (magnification 100×, 200×, black arrows: hemorrhage; green arrows: necrotic area; yellow arrows: inflammatory cell infiltration).** (B)** Infiltration of CD68+ cells was observed by immunohistochemistry (IHC). Representative staining of livers was performed using antibodies against the specific macrophage marker CD68 (magnification 200×). **(C)** Flow cytometric analysis of monocytes cells in liver tissues. The number of monocytes come from (CD45^+^ CD11b^+^) was showed in different groups. **(D)** Serum activities of ALT and AST were measured after 12 h (*P < 0.05, LPS+D-GAIN group versus Control group, ^#^*P*<0.05, L/D group versus L/D + MgTX group). **(E)** Serum levels of TNF-α was measured after 12 h (*P < 0.05, LPS+D-GAIN group versus Control group, ^#^*P*<0.05, L/D group versus L/D + MgTX group)

To examine the effects of MgTX on the inflammatory response in liver injury, ALI was induced in mice with LPS+D-GAIN (i.p.) and subjected to MgTX treatment. ALI and control mice were euthanized 12 h after LPS+D-GAIN or vehicle injection. Hematoxylin eosin (H&E) staining showed that LPS+D-GAIN injection caused significant ALI including hepatic steatosis, liver injury, and histological changes, which were significantly attenuated in MgTX treatment mice. In comparison, no injury was evident in the control group (Fig. 1A). Inflammatory cells infiltrate into liver was inhibited in mice compared with the control group, and MgTX (100 nM) could attenuate ALI in LPS+D-GAIN model and inhibit macrophage migration (CD68^+^) from peritoneal cavity to liver tissues (Fig. 1B), furthermore, special markers (CD45+, CD11b+, CCR2+, Gr1+) are used by flow cytometry to identify the CD68+ cells are infiltrating monocytes (Fig. 1C). Serum ALT and AST activities were significantly higher in LPS+D-GAIN-induced ALI mice, while MgTX treatment significantly decreased the serum levels of ALT, AST and TNF-α that were comparable to control groups (Fig. 1D, E). These results indicate the MgTX could attenuate ALI *in vivo*.

### 2. MgTX reduces the level of δ-catenin in macrophages by transcriptome analysis

**Figure 2:**
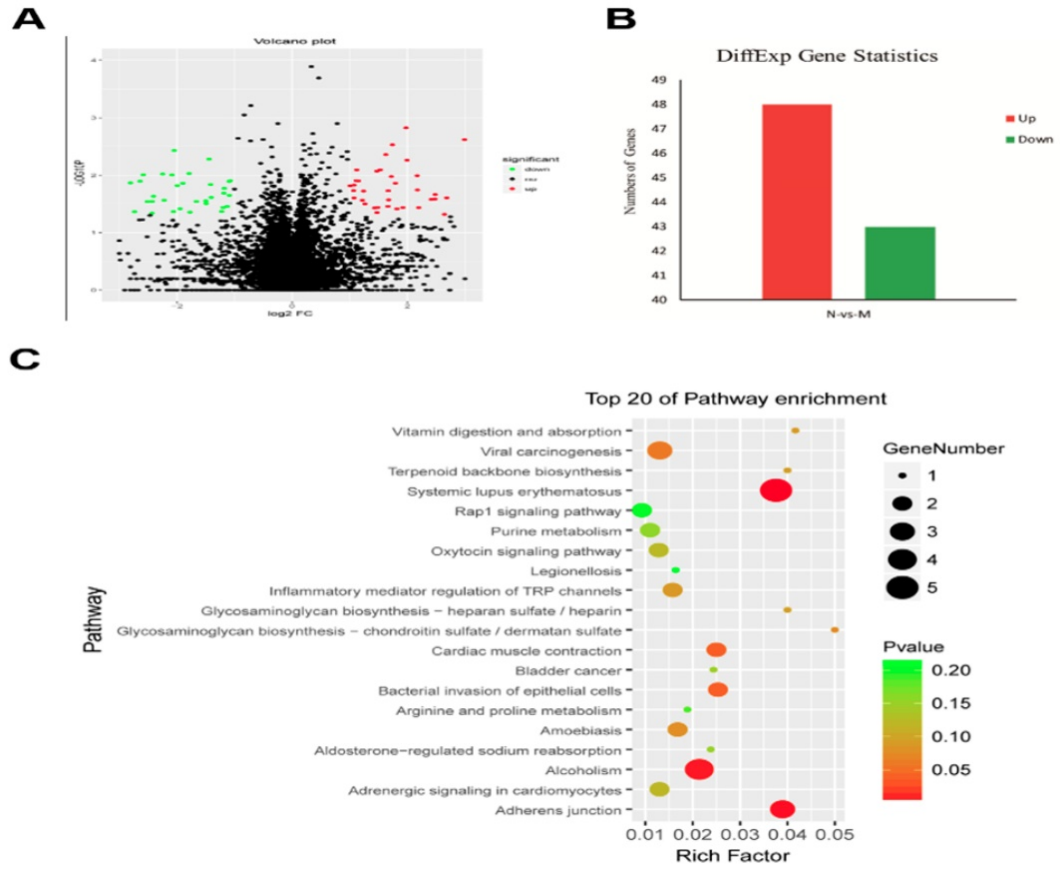
Lower levels of δ-catenin in MgTX-treated macrophages by transcriptome analysis. **(A)** Different genes were regulated by MgTX treatment as shown by RNA-seq in volcano plot. **(B)** Differential gene expression profile indicating that 48 DEGs were significantly upregulated and 43 DEGs were downregulated compared with control. **(C)** 20 DEGs associated pathways were affected by MgTX treatment.

To identify the genes related to the MgTX-induced Kv1.3 inhibition, we studied the transcriptome profile of RAW264.7 cell lines following treatment with MgTX (10 nM). The RNA-seq analysis is shown in a volcano plot (Fig. 2A). Functional annotation of the novel genes was performed in a BLAST search of public databases, including NCBI Nr and Nt database, Gene Ontology database, KOG database, SwissProt protein database, and KEGG database. Overall, 91 differentially expressed genes (DEGs) were enriched in MgTX-treated groups. Compared to the control group, 48 DEGs were significantly upregulated and 43 DEGs were downregulated. Minimum threshold standard of δ-catenin in MgTX-stimulated macrophages was selected by RNA-seq (*P* < 0.05 and |log2FC| > 1) (Fig. 2B). Furthermore, >20 DEGs associated pathways were found in MgTX treatment group, which involves δ-catenin modulation of cell adhesion function, such as Rap1 signaling and adherens junction pathways (Fig. 2C).

### 3. Expression of δ-catenin is downregulated by MgTX in a dose-dependent manner

**Figure 3:**
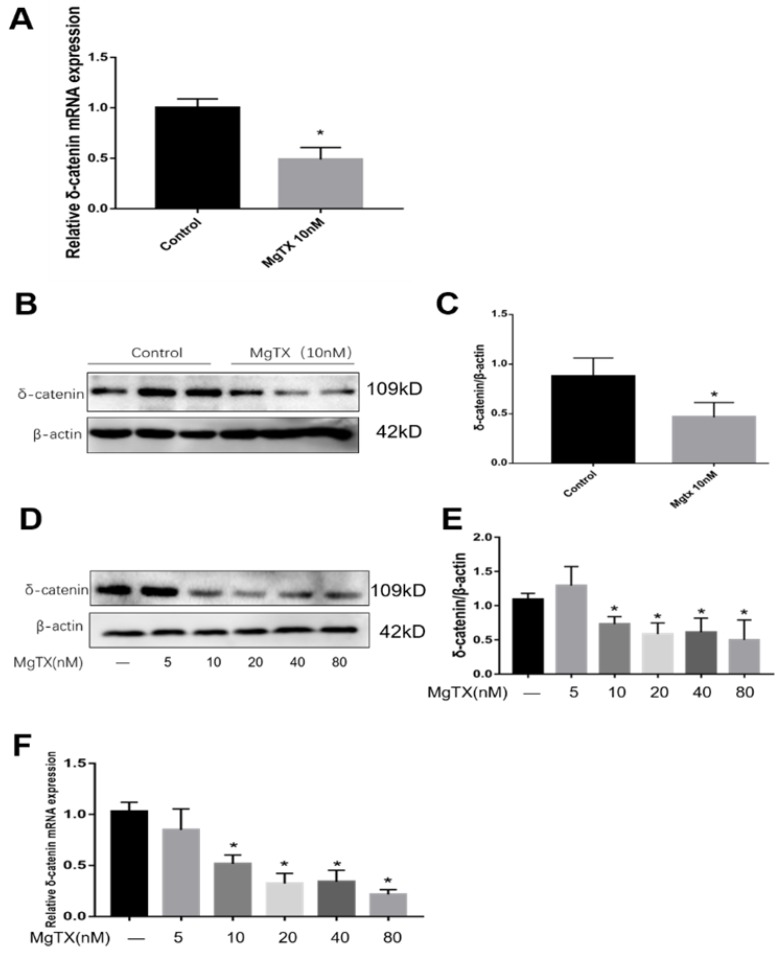
Expression of δ-catenin was downregulated by MgTX in a dose-dependent manner. After incubating for 12 h with or without MgTX, the mRNA and protein levels of δ-catenin in RAW264.7 cell lines were measured. **(A-C)** Expression of δ-catenin was inhibited by MgTX (10 nM) as shown by RT-qPCR and Western blot compared with control group (*P < 0.05). **(D-F)** Expression of δ-catenin was regulated by different concentrations of MgTX (0, 5, 10, 20, 40, and 80 nM) as shown by RT-qPCR and Western blotting compared with control group (*P < 0.05). There was no significant change at doses <5 nM.

We selected δ-catenin as the DEG of interest based on RNA-seq analysis. First, we examined whether MgTX (10 nM) could inhibit the expression of δ-catenin in RAW264.7 cell lines. As shown in Fig.3 (A-C), expression of δ-catenin was significantly inhibited in the presence of 10 nM MgTX at the transcriptional and protein levels as compared with control group. Furthermore, we investigated whether the relative degree of inhibition was dependent of the dose of MgTX (0, 10, 20, 40, and 80 nM). As shown in Fig.3 (D-F), as the concentration of MgTX increased to >10 nM, inhibition expression of δ-catenin was enhanced, while there was no significant change at doses <5 nM. This result was consistent with RNA-seq analysis and suggests that δ-catenin might be the target of MgTX.

### 4. Macrophage migration is inhibited by MgTX *in vitro*

**Figure 4:**
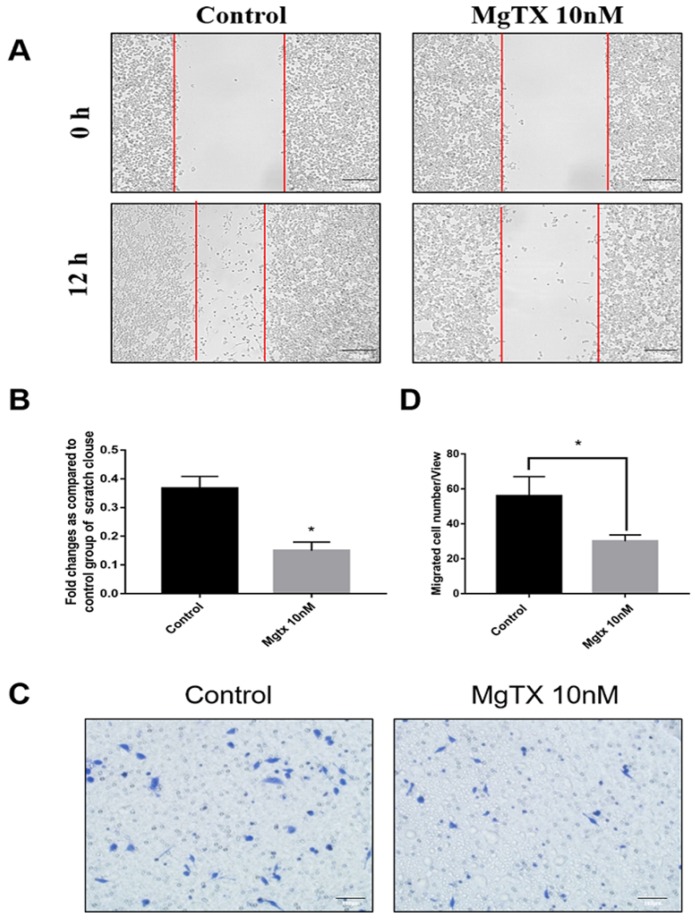
MgTX impairs migration of macrophages. Cells were incubated in the presence or absence of 10 nM MgTX for 12 h. **(A)** Migrating cells are found at the edge of the scratch and rate of migrating in cells treated with MgTX was slower than those in control group (magnification 200×). **(B)** Significant differences were found in macrophage migration in cells treated with 10 nM MgTX (*P < 0.05 vs. control). **(C)** MgTX inhibited macrophage migration crossing the transwell chamber and the number of migrating cells treated with MgTX in the upper chamber was less than that of control group. **(D)** Significant differences were found in macrophage migration treated with 10 nM MgTX (*P < 0.05 vs. control).

We examined the effects of MgTX on macrophage migration by performing cells wound healing and transwell assays. Representative figures of cell migration assays were shown in Figure 4(A-D), and the percentage of cell coverage to the scratch zone was analyzed and the number of migrating cells was counted with ImageJ software. For both the wound healing and transwell assay, macrophage migration at 12 h was blocked by MgTX (10 nM), as compared with control cells. This data suggested that MgTX could inhibit the migratory capacity of RAW264.7 cells.

### 5. Effect of δ-catenin expression on macrophage migration* in vitro*

**Figure 5:**
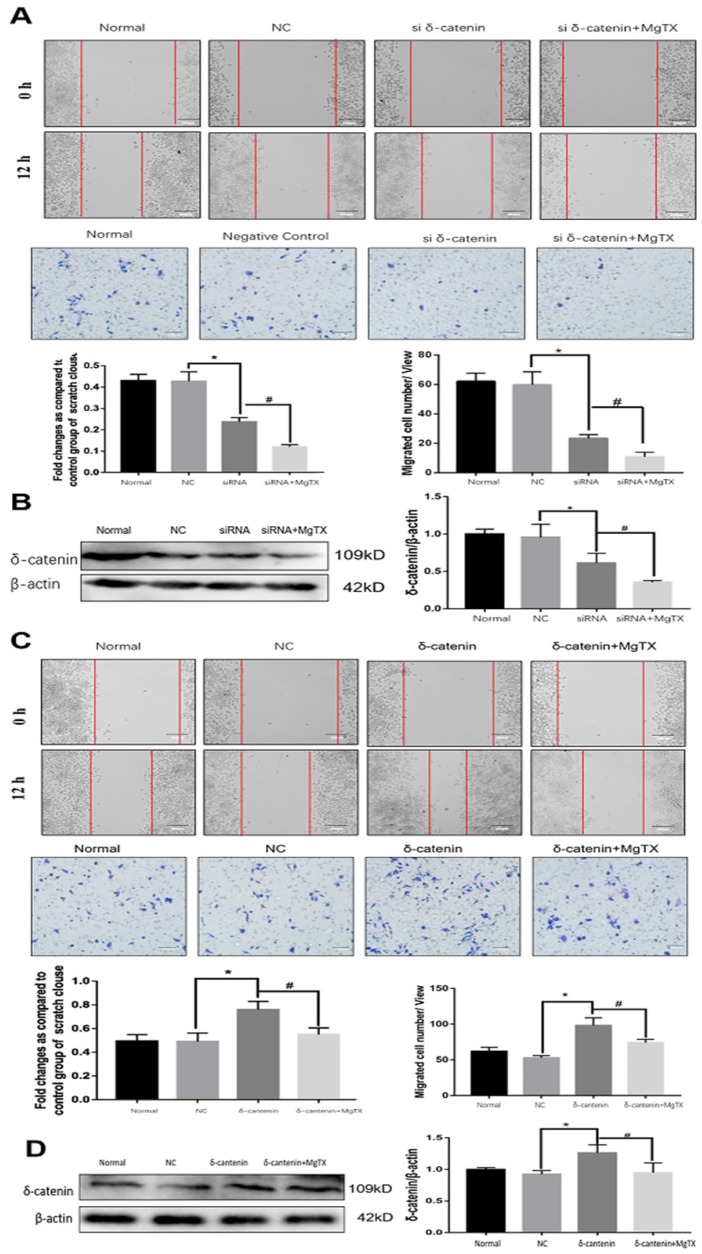
Macrophage migration is regulated by δ-catenin expression in RAW264.7 cells. **(A)** Comparison of macrophage migration in δ-catenin siRNA group, δ-catenin siRNA plus MgTX-treated group and control group. **(B)** Protein levels of δ-catenin expression were evaluated by Western blotting. **(C)** Comparison of macrophage migration in δ-catenin overexpression group, δ-catenin overexpression plus MgTX-treated group and control group. **(D)** Protein levels of δ-catenin expression were evaluated by Western blotting. Data are representative of three independent experiments; *P < 0.05.

We next investigated the impact of δ-catenin expression on the migration of RAW264.7 cell lines by performing wound healing and transwell assay. δ-catenin was knocked down or over expressed in RAW264.7 cell lines by transfection assays for 12 h. As shown in **Figure 5 (A)**, macrophage migration was inhibited by δ-catenin siRNA and the inhibition effect was enhanced by MgTX (10 nM) treatment. The efficiency and specificity of δ-catenin knockdown was confirmed by Western blotting in** Figure 5 (B)**. The expression of δ-catenin was downregulated by δ-catenin siRNA, and further inhibited with MgTX (10 nM) treatment. In contrast, as shown in** Figure 5(C)**, overexpression of δ-catenin effectively enhanced the migration of macrophages, compared with control RAW264.7 cells, while this enhancement was attenuated by MgTX (10 nM) treatment. The efficiency and specificity of δ-catenin overexpression was verified by Western blotting in **Figure 5 (D)**. The expression of δ-catenin was upregulated by δ-catenin overexpression and decreased with MgTX (10 nM) treatment.

### 6. Expression of RhoA is regulated by δ-catenin *in vitro*

**Figure 6:**
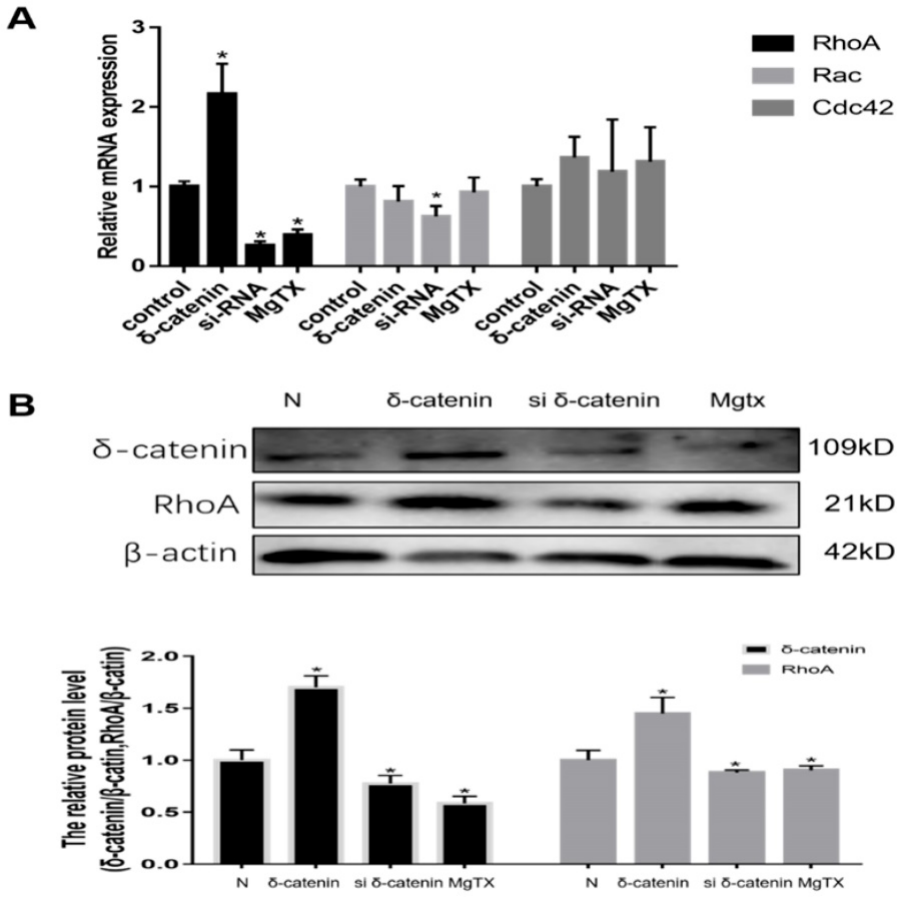
Expression of RhoA in RAW264.7 cell lines is regulated by δ-catenin knockdown, MgTX (10 nM) treatment, and δ-catenin overexpression. **(A)** Levels of RhoA, Rac, and Cdc42 mRNA were measured using qRT-PCR. **(B)** Expression of RhoA is regulated by δ-catenin knockdown, MgTX treatment, and δ-catenin overexpression as evaluated by qPCR and Western blotting. Data are representative of three independent experiments; *P < 0.05.

We next investigated the mechanism of δ-catenin regulation of macrophages migration and its downstream target in RAW264.7 cell lines. Previous RNA-seq results showed that Rho GTPases signal was downstream of δ-catenin. Therefore, we evaluated the expression of mRNA levels of Rho GTPases, including Rho A, Rac, and Cdc42, using qRT-PCR. As shown in **Figure 6 (A),** we found that the expression of Rho A was upregulated in δ-catenin-overexpressing cells and significantly inhibited in δ-catenin-knockdown cells. In comparison, the expression of Rac and Cdc42 had no obvious changes in response to alteration of δ-catenin expression. In further examination of the relationship between δ-catenin and Rho A, we found that the expression of Rho A was downregulated by δ-catenin loss and MgTX (10 nM) treatment, and upregulated by δ-catenin overexpression in RAW264.7 cell lines in Figure 6 (B).

In this study, we demonstrated that δ-catenin/Rho GTPases signaling pathway is involved in the modulation of migratory ability of RAW264.7 cells through regulating RhoA expression *in vitro*. This could be the underlying molecular mechanism by which δ-catenin regulates the migration of RAW264.7 cell lines.

## Discussion

Kv1.3 is a subtype of voltage-gated potassium channel, initially identified in human T lymphocytes^10^. Kv1.3 has an immunoregulatory function and is an important factor in regulating the activity and differentiation of T cells^11^. Blocking Kv1.3 can inhibit Ca^2+^ signaling, T cell proliferation, and IL-2 secretion 12, 13. While the role of the Kv1.3 channel has been established in regulating T cell function, few studies have examined its role in macrophages. Nonetheless, some of these previous studies have indicated the potential role that Kv1.3 may play in regulating macrophage function. Kv1.3 is indispensable for monocyte migration and inhibition of Kv1.3 can block monocyte chemotaxis and monocytes infiltration into injury brain. Furthermore, microglial activation and release of neurotoxic factors by activated microglial cells, including reactive oxygen species and pro-inflammatory cytokines occurs upon inhibition of Kv1.3^4^. Similarly, the infiltration of monocytes may play important roles in acute liver injury. Two types of macrophages that are important in ALI: the abundant liver-resident macrophages (“Kupffer cells”) and LY6Chi macrophages that are recruited from the bone marrow^14^, ^15^, what's more, infiltrating monocytes can be identified by their differential expression of the surface molecular Gr1 and the chemokine receptor CCR-2, evidence has showed specific contribution of CCR2-dependent Gr1^high^ monocytes in hemolytic uremic syndrome^16^. Our results demonstrated that the blocker of Kv1.3 channel prevent LPS-D-GAIN-induced liver damage by reducing excessive hepatic macrophage recruitment, which suggest that Kv1.3 channel may be a target for protecting ALI from immunological damage. Furthermore, if hepatocytes with MgTX treatment undergo apoptosis need to be clarified for further study, MgTX may attenuate acute liver injury through inducing apoptosis of hepatocyte not associated with macrophages infiltration.

Previous research and the data presented herein indicated that Kv1.3 could inhibit the macrophages migration; however, the mechanism of the Kv1.3 channel modulates macrophage migration is unclear. MgTX has 39 amino-acid-long peptide stabilized by three disulfide bridges with a molecular weight of 4185, and was extracted from the venom of the scorpion *Centruroides margaritatus*^17^. MgTX is a selective inhibitor of the Kv1.3 channel which binds it with high affinity. According our results, MgTX inhibits macrophage migration through reducing δ-catenin gene and protein expression. δ-Catenin (CTNND1) or neural plakophilin-related armadillo protein (NPRAP), belong to the member of β-catenin superfamily, which has 10 armadillo repeats (a 42-amino acid motif and firstly described in the Drosophila segment polarity gene, armadillo). These armadillo repeats can be spaced in the characteristic sequence of this gene family, including the prototypical member, p120ctn, p0071 and ARVCF, all of them contain a highly homologous repeating central armadillo domain^18^-^21^. β-catenin superfamily members interplay with cadherin through its central domain and are related to the actin cytoskeleton, then regulate the function of cell adhesion and process elaboration 8, 22-25. δ-Catenin is abundantly expressed in the brain, many evidences prove that appropriate expression of δ-catenin play crucial role in maintaining brain function. Targeted knockout of the *δ-Catenin* gene in mice leads to severe damage in cognitive function and short-term or long-term synaptic plasticity, which is associated with learning and memory of the brain 25. Ghose *et al*., detected the expression of δ-catenin in lymphatic endothelial cells, and those authors found that δ-catenin increased lymphangiogenesis* in vitro* and* in vivo*, and through modulation of small RhoGTPase activities. Consistent with inactivation of δ-catenin, lymphangiogenesis and growth of tumor metastases were inhibited in mouse models^26^. Nevertheless, research investigating δ-catenin function in regulating macrophages migration has been lacking. In this study, we used RNA-seq analysis and found that MgTX can downregulate δ-Catenin RNA and protein expression. Furthermore, macrophage migration was inhibited upon treatment with MgTX and transfection with δ-Catenin siRNA, while macrophage migration was increased upon δ-Catenin overexpression. These results showed that δ-Catenin plays a crucial role in regulating macrophage migration.

To further understand the essential role of δ-Catenin in mediating macrophage migration by RhoGTPase pathway, we investigated the impact on RhoGTPase family, which is located in downstream of δ-Catenin protein. Small Rho family of GTPase are related to cell motility, and previously studies identified that δ-catenin can regulate neuronal cell and vascular endothelial cell motility through Rho GTPase^27^. The mammalian Rho GTPase family currently contains seven different isoforms, including Rho (A, B, and C), Cdc42 (Cdc42Hs and G25K), Rac (1 and 2), RhoD, RhoE, RhoG and TC10. Similar to other members of Ras superfamily, Rho proteins act as molecular switches to control cellular processes by binding GTP or GDP 28. Rho, Rac, and Cdc42 are associated with function of cell motility, cytokinesis, axonal guidance and morphogenetic processes. In the present study, we found that δ-catenin silencing, overexpression and MgTX treatment altered the expression of RhoA. A positively correlated relationship was observed between δ-catenin and RhoA, while no obvious change was evident with Rac and Cdc42. These results suggested that RhoA might be an indispensable downstream protein in δ-catenin-mediated macrophage migration through the δ-catenin/RhoA pathway.

## Conclusion

In summary, we demonstrate for the first time that MgTX can alleviate ALI though inhibition of macrophage migration and the δ-catenin/RhoA pathway may be an important player in mediating this process. The current findings not only increase our understanding of how MgTX inhibits macrophage migration in ALI, but also suggest a new direction for development of potential targets in acute liver injury.

## Abbreviations

ALI: acute liver injury
Kv1.3: Voltage-gated potassium channel 1.3
Margatoxin: MgTX
ALF: Acute liver failure
KCs: Kupffer cells
LPS: lipopolysaccharide
IL-1: interleukin-1
IL-6: interleukin-6
MCP1: monocyte chemoattractant protein-1
TNF-α: tumor necrosis factor- α
D-GAIN: D-galactosamine
HE: hematoxylin - eosin
ALT: alanine aminotransferease
AST: aspartate aminotransferase
DMEM: Dulbecco's-modified Eagle's medium
I.P.: intraperitoneal
